# Performance of the Alice PDx Device With the Somnolyzer Automated Scoring Algorithm for the Diagnosis of Obstructive Sleep Apnea

**DOI:** 10.7759/cureus.52654

**Published:** 2024-01-21

**Authors:** David S Gomes, Carlos Seixas, João Cravo

**Affiliations:** 1 Pulmonology Department, Centro Hospitalar do Baixo Vouga, Aveiro, PRT; 2 Department of Research on Economics Management and Information Technologies, Portucalense University, Porto, PRT

**Keywords:** sleep apnea diagnosis, obstructive sleep apnea, apnea-hypopnea index, automated scoring algorithm, somnolyzer, alice pdx

## Abstract

Objective

Automated scoring of respiratory events could allow a swifter obstructive sleep apnea (OSA) identification. We assessed the accuracy of the Alice PDx device with the Somnolyzer automated scoring algorithm, compared to the manually reviewed scoring by a trained sleep technician, for the diagnosis of OSA.

Methods

A prospective study was conducted between March 2021 and March 2022 in Centro Hospitalar do Baixo Vouga, a level 2 hospital in Aveiro, Portugal. Patients with high pre-test probability for OSA performed a type III home sleep apnea testing with the Alice PDx device. Data were scored automatedly by the Sleepware G3 with the Somnolyzer digital system and manually by a trained sleep technician. Correlation and dependent t-tests were used. Sensitivity, specificity, positive predictive values (PPVs), negative predictive values (NPVs), and area under the receiver operating characteristic curve (AUROC) of automated scoring were calculated. Data were analyzed using the Stata Statistical Software (Release 17, StataCorp., 2023, College Station, TX: StataCorp LLC).

Results

In 150 participants (mean age 57.8 ± 13.9 years), the mean apnea-hypopnea index (AHI) was 21.9 ± 21.8 events/hour by manual scoring and 25.4 ± 21.6 events/hour by automated scoring. The mean difference was 3.4 ± 4.4 events/hour, and a strong, positive, linear correlation was found between the two scores (r = 0.98). At the altered AHI (AHI ≥ 5 events/hour), mild, moderate, and severe OSA, the automated scoring sensitivity/specificity values were 91.2%/100.0%, 80.0%/68.6%, 91.6%/41.9%, and 98.1%/80.9%, respectively. The PPVs/NPVs for the same categories were 100.0%/69.4%, 89.3%/51.1%, 79.7%/66.7%, and 91.8%/95.0%, respectively. Finally, the AUROC was 0.85, 0.70, 0.73, and 0.93, respectively.

Conclusion

The automated scoring obtained from the Alice PDx portable device, using Sleepware G3 with the Somnolyzer digital system, seems accurate enough to diagnose OSA and validate the initiation of PAP therapy in the correct clinical setting. Nevertheless, it does not replace manual reviewing by a trained sleep technician in the case of mild and moderate OSA, to obtain a correct severity classification. With this valuable time-saving tool, we expect to hasten OSA diagnosis and treatment and thus tackle the underdiagnosis problem.

## Introduction

Sleep-related breathing disorders comprise a broad spectrum of entities, including obstructive sleep apnea (OSA) [1]. OSA is defined by an apnea-hypopnea index (AHI) ≥15 with predominant obstructive respiratory events or an AHI ≥5 with predominant obstructive respiratory events accompanied by characteristic signs/symptoms [2]. OSA severity is classified using the AHI: mild (AHI = 5-14.9), moderate (AHI = 15-29.9), and severe (AHI ≥30) [3].

OSA affects mainly males and is closely related to increasing age and obesity. Its prevalence has increased in epidemiological studies over time [4], and it is estimated to occur in nearly one billion adults aged 30-69 years worldwide [5]. The only available study in the Portuguese population estimates OSA prevalence at 0.89% [6]. A large epidemiologic study in Hispanic/Latino individuals revealed that only 1.3% of participants reported a physician diagnosis of OSA, but OSA was present in 5.6% after conducting sleep apnea testing [7]. These data suggest that this condition is widely underdiagnosed.

If untreated, OSA contributes to a reduced quality of life and occupational and motor vehicle injuries [8]. More importantly, moderate-to-severe OSA is independently associated with a significantly increased risk of all-cause mortality [9].

Polysomnography (PSG) is still the standard diagnostic test for the diagnosis of OSA in adult patients [10]. Nevertheless, there is limited access to in-laboratory testing in some areas, where home sleep apnea testing (HSAT) is an alternative method that may be less costly and more efficient [10,11].

HSAT can be classified as type II, III, or IV depending on the measured parameters. In type III (or cardiorespiratory polygraphy), the most used HSAT, data are gathered through four to seven channels, measuring at least two respiratory variables (e.g., effort to breathe and airflow), oxygen saturation, and a cardiac variable (e.g., heart rate or electrocardiogram) [10]. In most cases, type III portable monitors have an algorithm for the automated scoring of events or allow data analysis by an automated digital system.

Given the increasing clinical use of HSAT [12], it would be important to determine the reliability and accuracy of automated scoring of events. Since the number of HSAT requests and subsequent waiting lists are increasing, the ability to produce an immediate report would be important. It would not only allow a swifter OSA identification but also significantly reduce the need for a manual review and scoring by a sleep technician, saving them time, and thus contributing to a reduction of OSA underdiagnosis [13]. Therefore, automated scoring could be a valuable solution when there is an urgency in treatment initiation and when the waiting time for a manual report is too long.

Our study intends to assess the accuracy of the Alice PDx portable device with the Somnolyzer automated scoring algorithm, compared to the manually reviewed scoring by a trained sleep technician, for the diagnosis of OSA. We also aim to find if and when a time-consuming manual review and scoring could be obviated.

## Materials and methods

Study sample

We performed an analytic observational prospective study in the pulmonology department of Centro Hospitalar do Baixo Vouga, a level 2 hospital in Aveiro, Portugal. The study lasted for 12 months, from March 2021 to March 2022.

We included adult patients who were observed in our department’s pulmonology consultation and had a clinical suspicion of OSA with a high pre-test probability. A high pre-test probability for OSA was defined as having a NoSAS score (neck circumference, body mass index, sex, age, snoring) of 8 or more [14] and/or a STOP-Bang score (Snoring, Tiredness during daytime, Observed apnea, High blood Pressure, Body mass index >35, Age >50, Neck circ >40 cm, Gender) of 5 or more [15]. We excluded patients with recording failures in any of the channels essential for recognizing respiratory events during sleep - oximetry, airflow, and respiratory effort - and those with recordings with fewer than four hours. Patients with a suspicion of insomnia, periodic limb movements, restless legs syndrome, or other sleep disorders unrelated to OSA were also excluded.

After enrolment, our patients were assigned to perform a type III HSAT with the Alice PDx device. Relevant clinical data (e.g., body mass index (BMI), age, sex, comorbidities, and Epworth sleepiness scale (ESS)) were collected. The automated HSAT report was placed into a specific folder. The manually reviewed report by a trained sleep technician was then added to that folder for data matching. A total of 150 patients were included.

The Ethics Committee of Centro Hospitalar Baixo Vouga, Aveiro, Portugal, approved the study (Ref. 214-CA-2-7).

Diagnostic procedures and equipment

The Alice PDx, a type II or III portable device, was used. In our study, only type III HSAT was performed, by recording the following signals: nasal pressure, snoring, rib cage and abdominal movement, body position, heart rate, and oxygen saturation by pulse oximetry. The type III HSATs were performed at home over the course of one night, and the equipment was returned to our pulmonology department the following day.

All physiologic data gathered through the Alice PDx portable device were collected and stored using the Sleepware G3 with the Somnolyzer digital system. This software performs fully automated data analysis and generates an automated report, according to the American Academy of Sleep Medicine (AASM) scoring criteria.

The manual review and scoring of raw data were completed according to the AASM scoring criteria by three trained sleep technicians, using the Sleepware G3 digital system. Sleep technician identification was not collected since it was not an objective to study inter-rater variability.

Statistical analysis

Data were analysed using the Stata Statistical Software (Release 17, StataCorp., 2023, College Station, TX: StataCorp LLC). Normally distributed continuous data variables were expressed as the mean ± standard deviation (SD). Pearson’s correlation was used to assess the relationship between automated and manual scoring of HSAT data (AHI, obstructive apnea index (OAI), central apnea index (CAI), and oxygen desaturation index (ODI)). The mean difference between HSAT data paired values was assessed using a dependent t-test. An agreement analysis between the AHI manual and automated scoring was assessed according to Park et al. [16]. Sensitivity, specificity, PPVs, NPVs, and AUROC of automated scoring were calculated for identifying altered AHI (AHI ≥5) and identifying OSA severity [17]. In this study, we adopted the AUROC values that Hosmer et al. recommended: 0.7 to 0.8 is considered acceptable, 0.8 to 0.9 is considered excellent, and 0.9 to 1.0 is considered outstanding [18]. A p-value of <0.05 was considered statistically significant.

## Results

A total of 150 participants were included. The mean age was 57.8 ± 13.9 years, 62.7% were male, and the mean BMI was 32.4 ± 6.2 kg/m^2^. Obesity was present in 66.0%, hypertension in 64.7%, diabetes in 31.3%, ischemic heart disease (with or without heart failure) in 29.3%, and depression in 25.3% of the participants.

The automated and manual scoring of HSAT data, paired correlations, and differences between paired values are presented in Table 1.

**Table 1:** Automated and manual scoring of HSAT data, paired correlations, and differences between paired values in the study sample AHI: apnea-hypopnea index; CAI: central apnea index; HSAT: home sleep apnea testing; OAI: obstructive apnea index; ODI: oxygen desaturation index; OSA: obstructive sleep apnea; SD: standard deviation

HSAT data	Manual scoring	Automated scoring	Pearson correlation coefficient		Paired differences: automated scoring - manual scoring	
	Mean ± SD - events/h	Mean ± SD - events/h	r	p	Mean ± SD - events/h	p
AHI	21.9 ± 21.8	25.4 ± 21.6	0.98	0.000	3.4 ± 4.4	0.000
OAI	10.3 ± 15.6	8.2 ± 9.5	0.87	0.000	(-) 2.1 ± 8.8	0.004
CAI	2.5 ± 6.0	2.2 ± 4.2	0.88	0.000	(-) 0.3 ± 3.1	0.192
ODI	28.4 ± 25.0	28.4 ± 25.0	1.00	0.000	0.0 ± 0.0	0.000

The OSA severity classification and respective paired differences between the AHI automated and manual scorings are presented in Table 2.

**Table 2:** OSA severity classification and respective paired differences between the AHI automated and manual scorings in the study sample AHI: apnea-hypopnea index; OSA: obstructive sleep apnea; SD: standard deviation

OSA severity classification	Manual scoring	Automated scoring	Paired differences: AHI automated scoring - AHI manual scoring	
	n (%)	n (%)	Mean ± SD - events/h	p
Normal	36 (24.0%)	25 (16.7%)	1.4 ± 1.4	0.000
Mild	47 (31.3%)	35 (23.3%)	5.1 ± 3.1	0.000
Moderate	27 (18.0%)	43 (28.7%)	5.3 ± 5.4	0.000
Severe	40 (26.7%)	47 (31.3%)	2.0 ± 5.5	0.024

**Figure 1:**
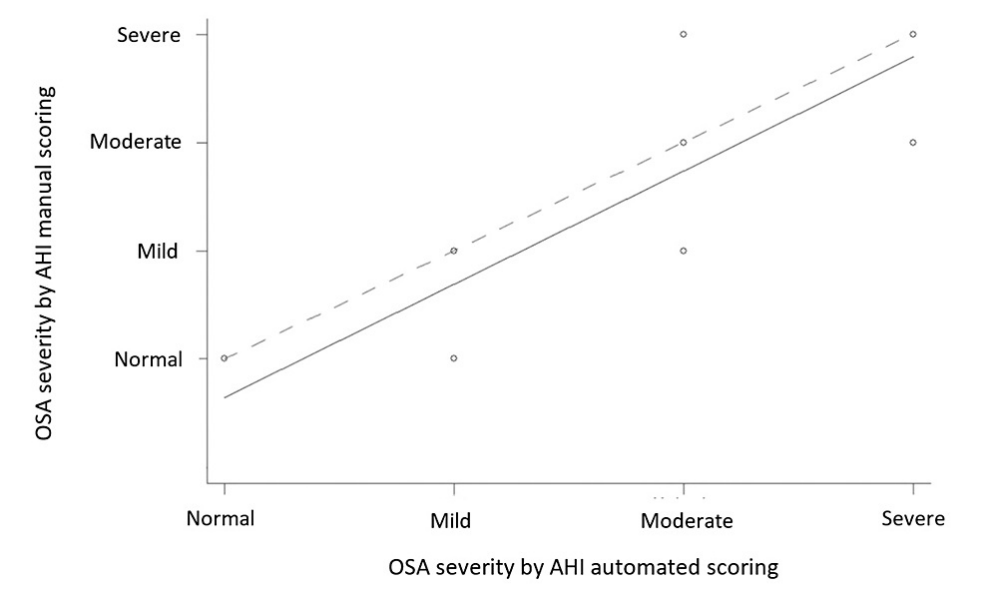
Plot showing the correlation coefficient of agreement between the AHI automated and manual scoring AHI: apnea-hypopnea index; OSA: obstructive sleep apnea

AHI automated scoring trended toward overestimation. Using the correlation coefficient of agreement, the agreement between the AHI manual and automated scoring is positive and significant (p = 0.000) for both the asymptotic case and the z-transformation. In Figure 1, we can verify that divergences between the AHI manual and automated scoring occur for values of mild OSA severity (AHI <15).

The sensitivity, specificity, PPV, and NPV of the automated scoring for identifying OSA and the OSA severity are shown in Table 3.

**Table 3:** Sensitivity, specificity, PPV, and NPV of the automated scoring for identifying OSA and the OSA severity AHI: apnea-hypopnea index; NPV: negative predictive value; OSA: obstructive sleep apnea; PPV: positive predictive value

OSA severity classification	Sensitivity	Specificity	PPV	NPV
Altered AHI (AHI ≥ 5)	91.2%	100.0%	100.0%	69.4%
Mild (AHI 5 – 14.9)	80.0%	68.6%	89.3%	51.1%
Moderate (AHI 15 – 29.9)	91.6%	41.9%	79.7%	66.7%
Severe (AHI ≥30)	98.1%	80.9%	91.8%	95.0%

**Figure 2:**
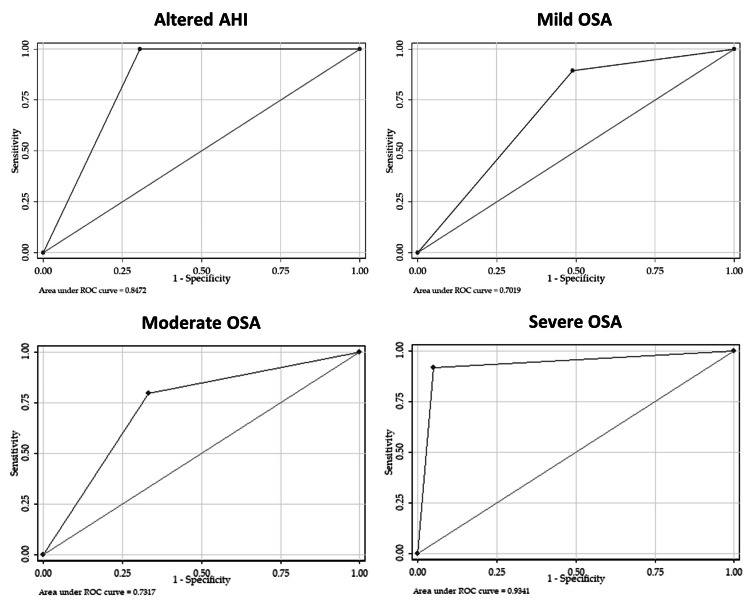
Comparisons of the automated scoring AUROC curves for identifying OSA and the OSA severity AHI: apnea-hypopnea index; AUROC: area under the receiver operating characteristic curve; OSA: obstructive sleep apnea

Figure 2 shows the comparisons of automated scoring AUROC curves for identifying OSA and the OSA severity. At altered AHI (AHI ≥5 events/hour), mild, moderate, and severe OSA, the AUROC values were 0.85, 0.70, 0.73, and 0.93, respectively.

ESS was only valid in 121 participants. Depression was present in 24.0% of these patients. Table 4 shows a mild, positive, linear correlation between ESS and AHI manual scoring (r = 0.22; p = 0.016). This correlation remained significant (p = 0.014) despite introducing the “depression” variable, although the presence of depression negatively influenced AHI manual scoring (p = 0.078). The ESS AUROC for identifying OSA was 0.51.

**Table 4:** ESS, AHI manual scoring, and respective correlations in the group of patients with a valid ESS questionnaire AHI: apnea-hypopnea index; ESS: Epworth sleepiness scale; SD: standard deviation

	ESS	AHI manual scoring	Pearson correlation coefficient		Pearson correlation coefficient (after introducing the “depression” variable)	
	Mean ± SD - score	Mean ± SD - events/h	r	p	r	p
Valid ESS group (121 patients)	10.2 ± 5.5	23.2 ± 23.0	0.22	0.016	0.22	0.014

## Discussion

Automated scoring showed excellent discrimination (AUROC = 0.85) and sensitivity for identifying altered AHI (AHI ≥ 5) and a perfect specificity and positive predictive value, indicating its great potential use as a screening tool for OSA, in patients with high pre-test probability.

For severe OSA identification, automated scoring showed outstanding discrimination (AUROC = 0.93), excellent sensitivity, good specificity, and a low mean difference between AHI paired values, indicating that there may be no need for manual review and scoring in such cases.

As for mild and moderate OSA identification, automated scoring showed lower sensitivity and specificity values, only acceptable discrimination (AUROC = 0.70 and 0.73, respectively), and a higher mean difference between AHI paired values, indicating that there may be the need for manual review and scoring in such cases.

These study results demonstrate that the Alice PDx automated scoring using Sleepware G3 with the Somnolyzer digital system provides an accurate OSA diagnosis in the correct clinical setting. We suggest that it can be used to validate the initiation of positive airway pressure (PAP) therapy in symptomatic patients with a high pre-test probability of OSA. Nevertheless, a correct severity classification in the case of mild and moderate OSA requires manual review and scoring. Finally, a PSG should be conducted if both AHI automated and manual scoring is <5 events/hour and high clinical suspicion persists, given the possibility of AHI underestimation.

These results are concordant with studies using other commercially available automated scoring systems, also showing a strong agreement in the scoring of the AHI for HSATs between the automated scoring system and experienced sleep technicians [13].

Nilius et al. demonstrated that using the manually scored Alice PDx portable device in cases with a high pretest OSA probability had a high level of diagnostic agreement with a simultaneous PSG and performed valid home diagnostic studies [19]. Still, no other studies tried to validate its automated scoring.

Anderer et al. and Punjabi et al. demonstrated the validity of the Somnolyzer automated scoring algorithm for PSG [20,21]. Nonetheless, to our knowledge, no other study tried to validate it for data collected from a type III HSAT.

A strong correlation and a non-significant mean difference between CAI paired values were found, indicating the possible utility of this HSAT device for central sleep apnea syndrome screening. Nevertheless, CIA in our population was <5 events/hour and there was no clinical suspicion, so a target population-based study must be conducted.

ESS was not useful in predicting the occurrence of OSA, matching the results of other studies [22,23]. The presence of depression is important when evaluating ESS since excessive daytime sleepiness is frequent in these patients.

Reducing the time and resources needed to obtain an OSA diagnosis is crucial to tackling underdiagnosis. Alice PDx may serve as a simple, rapid, and accessible screening device since it allows automated scoring, but it also permits manual review and scoring of the raw data by a sleep technician. As in PSG, good quality of the HSAT recording is mandatory for either automated or manual scoring to be reliable.

By expanding the range of accurate automated scoring algorithms that can be used, we expect to help hasten OSA diagnosis and lessen the underdiagnosis problem.

The main limitation of this study is that HSAT data were not confirmed by PSG, the gold standard for OSA diagnosis, which may have resulted in an underestimation of the true AHI. Since we studied a group of patients with a high pre-test probability of OSA, these findings cannot be applied to the general population. In addition, the single centre location and the low number of scoring technicians may have the potential for bias. Finally, our results cannot be extrapolated to other commercially available automated digital systems.

## Conclusions

Automated scoring obtained from the Alice PDx portable device, using Sleepware G3 with the Somnolyzer digital system, seems accurate enough to diagnose OSA and validate the initiation of PAP therapy in the correct clinical setting. Nevertheless, it does not replace manual reviewing by a trained sleep technician in the case of mild and moderate OSA to obtain a correct severity classification.

Automated scoring may be particularly beneficial when there is an urgency in treatment initiation or when waiting times for manual reports are prolonged. With this valuable time-saving tool, we expect to hasten OSA diagnosis and treatment and thus tackle the underdiagnosis problem.
